# Plasma membrane receptor mediated MAPK signaling pathways are activated in human uterine cervix at parturition

**DOI:** 10.1186/1477-7827-5-3

**Published:** 2007-01-28

**Authors:** Hong Wang, Ylva Vladic Stjernholm

**Affiliations:** 1 Division for Reproductive Endocrinology, Karolinska University Hospital and Karolinska Institutet, SE-171 76 Stockholm, Sweden; 2 Division for Obstetrics and Gynecology, Department of Woman and Child Health, Karolinska University Hospital and Karolinska Institutet, SE-171 76 Stockholm, Sweden; 3 Department of Cell and Molecular Biology, Karolinska University Hospital and Karolinska Institutet, SE-171 76 Stockholm, Sweden

## Abstract

**Background:**

Cervical ripening resembles an inflammatory reaction. Estrogens induce leukocyte migration into tissue and factors promoting cervical remodeling and labor, although the mechanisms are only partially known. The aim of this study was to investigate whether plasma membrane receptor mediated pathways, known to be activated by estrogens and proinflammatory compounds, are involved in cervical ripening before labor.

**Methods:**

The expression and distribution of mitogen activated protein kinases (MAPK), which transduce extracellular signals into intracellular responses through phosphorylation, and their intracellular targets transcription factors c-Jun and c-Fos proteins (AP-1) were analysed in cervical biopsies from term pregnant women (TP), immediately after parturition (PP), and from non-pregnant women (NP). Immunohistochemistry and RT-PCR techniques were used.

**Results:**

Cell-specific alterations in the immunostaining pattern for MAPK were observed. The expressions of activated, phosphorylated MAPK forms pERK1/2, pJNK and p38MAPK were significantly increased in cervical stroma until TP and pERK1/2 expression was significantly enhanced in PP group. c-Jun was significantly increased in cervical stroma and smooth muscle in TP as compared to NP group. c-Fos was significantly increased in stroma, squamous epithelium and glandular epithelium in PP as compared to TP group.

**Conclusion:**

We report, for the first time, cell-specific activation of pMAPKs and their targets transcription factors c-Fos and c-Jun (AP-1) proteins in human uterine cervix until term pregnancy, and immediately after parturition. These results suggest a role for MAPK activation in cervical ripening before labor.

## Background

Cervical remodeling is a prerequisite for effacement and dilatation prior to labor and is characterized by an increase in vascular adhesion molecules, diapedesis and activation of neutrophils, T-cells, monocytes/macrophages, mast cells, eosinophils, and increased concentrations of metalloproteinases [1-5].

Metalloproteinase enzymes effectuate an altered proteoglycan composition and increased collagen solubility, which allows for extracellular matrix remodeling and an increased cervical distensability [5-8]. Maternal plasma levels of estrogens increase exponentially until term pregnancy and at labor concomitant with unchanged or decreased progesterone levels [7,9,10]. The exact mechanisms leading to parturition in humans remain unclear, although previous studies have proposed that estrogens may contribute to extracellular matrix remodeling and inflammatory changes [1,10-12]. The suggested effects of estrogens during cervical ripening appear to be highly cell-type specific [11,12].

Mitogen activated protein kinases (MAPK) comprise proline-directed second messenger systems, used by eukaryotic cells for the transduction of extracellular signals into responses in the cytosol, nucleus and mitochondria through protein phosphorylation [13-16]. Factors eliciting MAPK signal transduction are identified including proinflammatory cytokines, growth factors, estrogens, and mitochondrial reactive oxygen species (ROS) e.g. thioredoxin [14-17]. The most well-characterized MAPK cascade consists of extracellullar signal-regulated (ERK) kinases MEK1/2 and ERK 1/2 (p42/p44), stress-activated protein kinases (SAPK) i.e. c-Jun amino-terminal kinase (JNK) and reactivating protein kinase (p38MAPK) [13-15]. Activation of MAPKs is mediated by upstream phosphorylation via MAPK kinases (MAPKKs) [14,15,18,19]. Once activated, MAPKs predominantly regulate gene expression via phosphorylation of downstream transcription factors, which include the proinflammatory transcription factors activator protein (AP)-1 and nuclear factor (NF)-κB [15,16,19,21,22]. AP-1 is a collective term referring to heterodimers formed by c-Jun, c-Fos or activating transcription factor subunits which bind to AP-1 responsive elements in gene promoters [17-20]. Despite the high maternal plasma estradiol level present at term pregnancy, the total nuclear estrogen receptor (ER) level in the uterine cervix is markedly decreased as compared to non-pregnant, suggesting a markedly limited nuclear ER mediated biological response to estrogens at parturition [7,9]. The aim of this study was to investigate the expression and distribution of MAPKs and their intracellular target transcription factor AP-1.

## Materials and methods

### Patients

The non-pregnant (NP) group consisted of healthy, regularly menstruating women (n = 6) without medication, undergoing hysterectomy due to benign disorders not involving the cervix. Hysterectomies were carried out during the follicular phase of the menstrual cycle within ten days from the latest menstrual period, to avoid the risk for an unknown pregnancy. The study subjects had a mean age of 42 years (range 32–49), and a mean parity of 2 (1–3). The term pregnant (TP) group consisted of healthy women (n = 8) with a mean age of 32 years (range 28–38), a mean gestational age of 38 weeks (range 37–39), and a mean parity of 1 (range 1–2). All had unripe cervices as determined by a Bishop score of < 5 points and none of them was in labor. Elective caesareans on medical indications were carried out in all women. The postpartum (PP) group consisted of primiparous, healthy women (n = 9) with uncomplicated pregnancies, from whom biopsies were obtained within 20 minutes after uncomplicated vaginal parturition. They had a mean age of 30 years (range 23–37), and a mean gestational age of 40 weeks (range 39–41).

Every woman had given her informed consent before entering the study. The study was approved by the Karolinska Hospital Ethics Committee (96–187, 99–099).

### Sampling procedure

The biopsies were obtained transvaginally from the anterior part of the uterine cervix at the 12 o'clock position, from 10–20 mm depth. The cervical biopsies were divided and one half was immersion-fixed in 4 % formaldehyde at 4°C overnight, stored at 4°C in 70% ethanol and thereafter embedded in paraffin. One half was immediately frozen at -70°C.

### Immunohistochemistry

Paraffin sections (5 μm) were used as a standard immunohistochemical technique (avidin-biotin-peroxidase) which was carried out as described before to visualize MAPK (p42/44-ERK1/2, p38 and JNK) immunostaining intensity and distribution [23]. Primary antibodies used in this study are listed in Table 1. All antibodies were incubated at 4°C overnight. Replacement of the primary antibody with an equivalent concentration of non-immune mouse IgG (for monoclonal antibody) and rabbit IgG (for polyclonal antibody) was used for negative controls.

**Table 1:** Primary antibodies used in this study.

**Antibody**	**Specificity**	**Dilution**	**Sources**
c-fos (Ab-2) Polyconal (PC05)	Amonio acid residues 4–17 of human c-Fos	1:40	Oncogene Research Products, Cambridge, MA
c-Jun (Ab-2) Polyclonal (PC07)	Amonio acid residues 91–105 of human c-Jun	1:40	Oncogene Research Products, Cambridge, MA
Phopho-specific P44/42 MAPK polyclonal Ab (9101S)	Amonio acid residues 91–105 of human p44 MAP kinase, detects p42 and p44 MAPK (Erk1 and Erk2), only when activated by phosphorylation at Thr 202 and Tyr 204	1:250	New England Biolabs, Inc. Beverly, MA
P44/42 MAPK polyclonal Ab (9102)	Amonio acid residues 345–358 of rat p42 MAPK, detects total MAP kinase (Erk1 and Erk2) levels	1:1000	New England Biolabs, Inc. Beverly, MA
Phospho-p38 MAPK polyclonal Ab (9211S)	A synthetic doubly phosphorylated peptide (Thr 180/Tyr 182) of human p38 MAPK	1:500	New England Biolabs, Inc. Beverly, MA
p-JNK (G-7) Monoclonal AB (sc-6254)	A short aa sequence containing phosphorylated on Thr-183 and Tyr-185 of JNK1 of human, reacts to phosporylated JNK 1–3	1:250	Santa Cruz Biotechnology, Inc. USA
JNK2 (D-2) Monoclonal AB (sc-7345)	Full lengh (aa 1–424) human JNK2, reacts to JNK1-3	1:250	Santa Cruz Biotechnology, Inc. USA

### Immunostaining analysis

A Leica microscope and Sony video camera (Park Ridge, NJ, USA) connected to a computer (Leica Imaging System Ltd, Cambridge, UK) was used to assess immunohistochemistry results. Two sections of each sample were analysed and ten areas of cell compartment from each section were randomly chosen for quantification of immunostaining intensity. The staining intensity was semiquantitatively graded (H.W.) by manual scoring as very strong (4) when 80–100% cells were positive, strong (3) when 60–80% of cells were positive, moderate (2) when 30–60% of cells were positive, weak (1) when less than 30% of cells were positive or as no staining (0).

### RNA isolation and RT-PCR analyses

Frozen pieces of cervical tissue were processed for isolation of total RNA according to the manual for the SV total RNA isolation system (Promega, Madison, WI, USA). One μg of total RNA were reversely transcribed at 42°C for 45 min in a final volume of 40 μl with a reaction mixture containing 50 mM Tris-HCl pH 8.3, 75 mM KCl, 3 mM MgCl_2_, 7.5 mM DTT, 0.5 mM dNTPs, 1 μg random hexamers and 400 IU of ML-MTV reverse transcriptase (GIBO, BRL, Paisley, UK). Primer sequences and the predicted product size were for human c-fos gene: 5'-GGATAGCCTCTCTTACTACCAC-3'; 5'-TCCTGTCATGGTCTTCACAACG-3' and 280 bp; the human c-jun gene: 5'-GGAAACGACCTTCTATGACGATGCCCTCAA-3'; 5'-GAACCCCTCCTGCTCATCTGTCACGTTCTT-3' which amplify a 325 bp fragment and the human ribosomal protein S28 which was used as an internal control: 5'-GTGCAGATCTTGGTGGTAGTAGC-3'; 5'-AGAGCCAATCCTTATCCCGAAGTT-3' and 552 bp. For PCR amplification, the cDNA (corresponding to 50 ng RNA from the RT-PCR reaction) was added to the reaction mixture containing 20 mM Tris-HCl pH 8.4. 50 mM KCl, 2.5 IU *Tag *DNA polymerase (GIBCO, BRL, Paisley, UK), 0.2 mM dNTPs, 1.5 mM MgCl_2_, and oligonucleotide primer pairs (50 pmol/pair), with a final volume of 50 μl. PCR was performed for 30 cycles using 94°C (30 s) for denaturing, 58°C (45 s) for annealing, 72°C (45 s) for extension and a final incubation at 72°C for 3 min on a thermal cycler. The products were subjected to electrophoresis in 2.5 % agarose gels; data were analyzed using a Fujifilm Las-1000 system (Fujifilm, Japan). Statistical analyses were not performed on this data.

### Statistical analyses

Statistical calculations for the immunohistochemistry results by image analysis of pMAPK were performed by ANOVA on ranks (Kruskal-Wallis test) and significances were evaluated by Dunn's test. One-way parametric ANOVA was used for analyses of immunohistochemistry results for c-Jun and c-Fos proteins.

## Results

**Figure 1:**
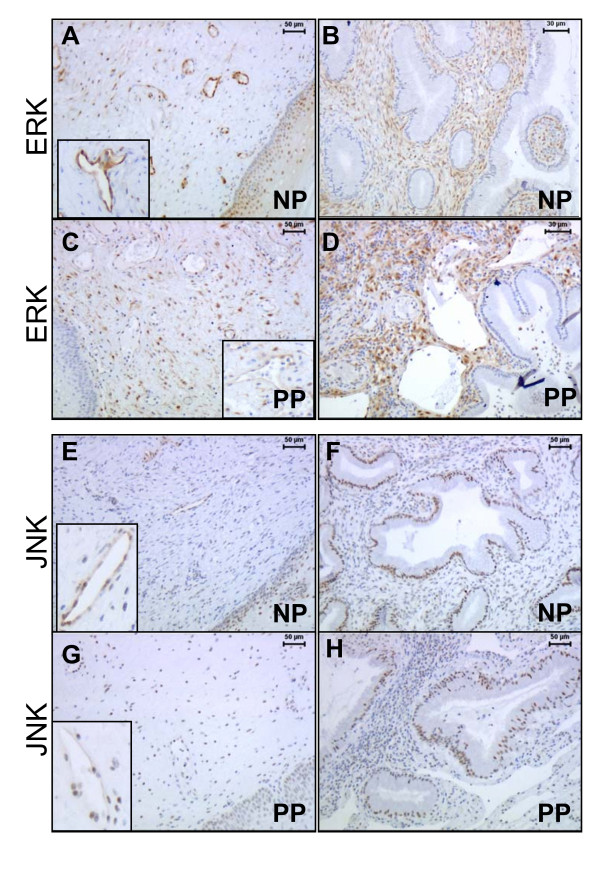
Representative sections showing the expression and distributions of pERK and pJNK in different cell types of the uterine cervix by using phospho-antibodies, as shown in non-pregnant (NP) and postpartum (PP) groups. Cervical stroma, blood vessels shown in A, C, E, G. Glandular epithelium and subepithelial mucosa area of stroma shown in B, D, F and H.

**Figure 2:**
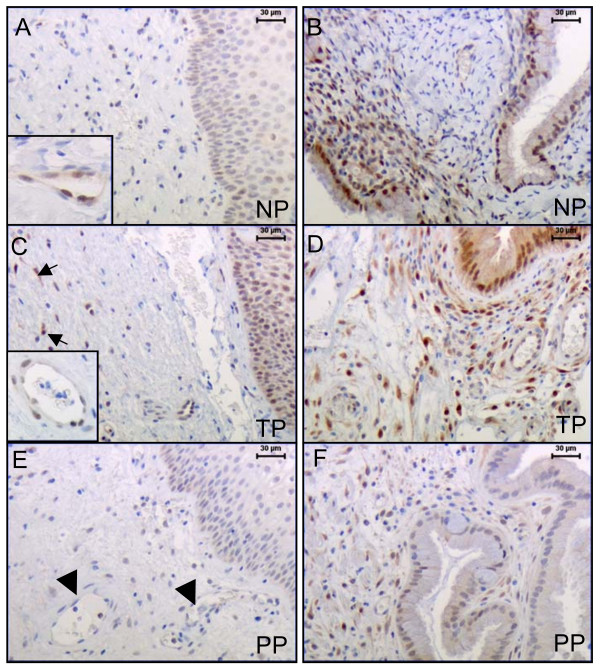
Representative cervical sections from non-pregnant (NP) (A-B), term pregnant (TP) (C-D) and postpartum (PP) groups (E-F), showing the the expression and distribution of p38MAPK in different cells by using phospho-antibodies. Stroma and blood vessels (A, C and E). Glandular epithelium and subepithelial mucosa area of stroma (B, D and F).

All three types of phosphorylated MAPKs were positively stained in the cervix, in both cell nuclei and cytoplasm (Figure 1 and 2). The distribution of pMAPKs by using different antibodies in the cervical connective tissue was described as following:

### ERK1/2 (p42/44)

**Figure 3:**
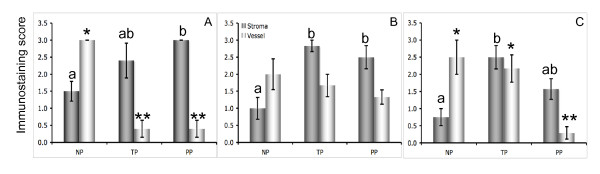
The immunostaining scores for pERK1/2 (A), pJNK (B) and p38MAPK (C) in cervical stroma and vascular endothelium in biopsies from non-pregnant (NP, n = 6), term pregnant (TP, n = 8) and postpartum (PP, n = 9) groups respectively. For stroma figures with different letter designation are significantly different (P < 0.05). For blood vessels significant values are marked with * for P < 0.05, and with ** for P < 0.01 respectively.

ERK immunostaining was observed in different compartments of the cervix by using non-phosphorylated and phosphorylated (pERK1/2) antibodies. The staining intensity was slightly higher by the non-phosphorylated antibody (data not shown), although the staining patterns did not differ significantly by these two types of antibodies. In cervical stroma, pERK1/2 expression was the lowest in the NP group, and increased in TP and PP groups as compared to NP (P < 0.05) (Figure 1A–D and Figure 3A). In the subepithelial mucosa area, intensive immunostaining was present in the stromal cells in all study groups. In smooth muscle, the pERK1/2 expression was unchanged between the groups (data not shown). The endothelial cells of blood vessels (vein) displayed high pERK1/2 expression in NP group, but weak or absent in TP and PP groups as compared to NP (P < 0.01) (Figure 1A,C and Figure 3A). No immunostaining for pERK 1/2 was found in glandular epithelium (Figure 1B,D).

### JNK

The phosphorylated JNK (pJNK) immunostaining was mainly localized to the cell nuclei, whereas non-phosphorylated JNK staining was found in both cytosol and nuclei (data not shown). In cervical stroma, pJNK expression was the lowest in the NP group, but significantly increased and high in TP and PP groups as compared to NP (P < 0.05) (Figure 1E–H and Figure 3B). In the subepithelial mucosa area, pJNK staining was present in some stromal cells in all study groups. High intensity pJNK staining was generally found in glandular epithelium but without significant changes between the study groups (Figure 1F,H). Vascular endothelial pJNK expression did not differ between the groups (Figure 1E,G and Figure 3B).

### p38MAPK

Only the phosphorylated-specific antibody for p38MAPK was used. A reciprocal pattern för p38MAPK expression was observed in cervical stroma in comparison to vascular endothelium between NP and TP groups (Figure 3C). High intensity p38MAPK immunostaining was present in the vascular endothelium in NP and TP groups and absent in PP group (P < 0.01) (Figure 2A,C (magnification), E (arrow head), and Figure 3C). p38MAPK expression in stroma was significantly increased in TP as compared to NP group (P < 0.05) (Figure 2 and Figure 3C). In the subepithelial mucosa area, p38MAPK staining was present in stroma cells in all study groups, but less intensive in the PP group (Figure 2B,D,F).

### c-Jun

**Figure 4:**
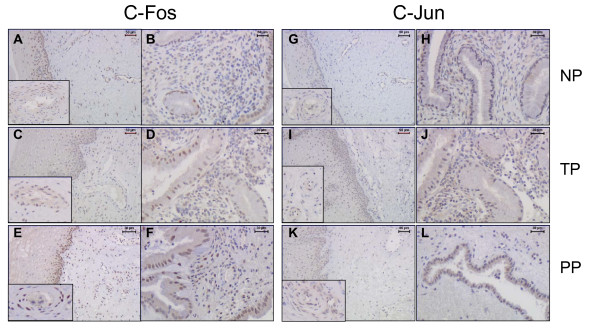
Representative sections showing the expression and distributions of c-Fos and c-Jun proteins (AP-1) in different cell types of cervical samples from non-pregnant (NP), term pregnant (TP) and postpartum (PP) groups. Stroma and blood vessels for c-Fos shown in in A, C, E and for c-Jun shown in G, I, K (magnification vein). Glandular epithelium and subepithelial mucosa area of stroma, shown for c-Fos in B, D, F and for c-Jun in H, J, L.

**Figure 5:**
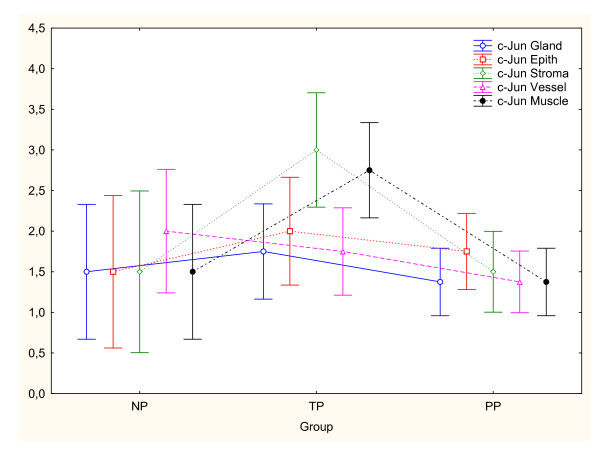
The immunostaining scores for c-Jun in different cell types in cervical biopsies from non-pregnant (NP, n = 2), term pregnant (TP, n = 4) and postpartum (PP, n = 8) groups. c-Jun immunoreactivity was significantly increased in cervical cervical stroma (P < 0.01) and smooth muscle (P < 0.001) in TP as compared to NP group. Changes in vascular endothelium, squamous epithelium and glandular epithelium did not differ between the groups. Wilks lambda = 0.07. F_10,16 _= 4.03.

Immunoreactivity for c-Jun protein was present in all cellular compartments in NP, TP and PP groups (Figure 4). c-Jun expression was significantly increased in cervical stroma (P < 0.01) and smooth muscle (P < 0.001) in TP as compared to NP group (Figure 5). The changes in vascular endothelium, squamous epithelium and glandular epithelium did not differ between the groups.

### c-Fos

**Figure 6:**
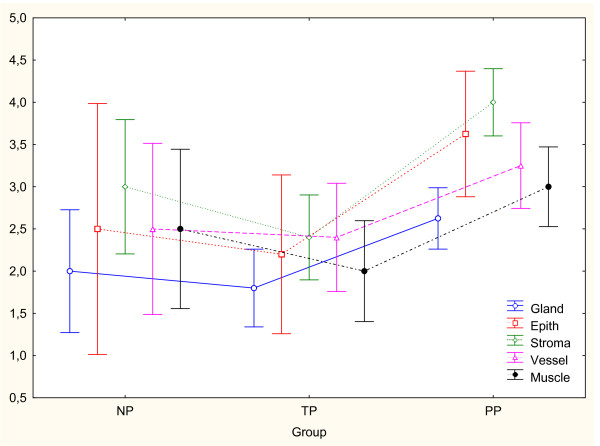
The immunostaining scores for c-Fos in different cell types in cervical biopsies from non-pregnant (NP, n = 2), term pregnant (TP, n = 4) and postpartum (PP, n = 8) groups. c-Fos immunoreactivity was significantly increased in cervical stroma (P < 0.01), squamous epithelium (P < 0.01) and glandular epithelium (P < 0.05) in PP as compared to TP group. Changes in vascular endothelium and smooth muscle did not differ between the groups. Wilks lambda = 0.14. F_10,16 _= 2.70, P > 0.05.

Immunoreactivity for c-Fos protein was present in all cellular compartments in NP, TP and PP groups, as shown in Figure 4. c-Fos expression was increased in cervical stroma (P < 0.01), squamous epithelium (P < 0.01) and glandular epithelium (P < 0.05) in PP as compared to TP group (Figure 6). The changes in vascular endothelium and smooth muscle did not differ between the groups.

### RT-PCR

**Figure 7:**
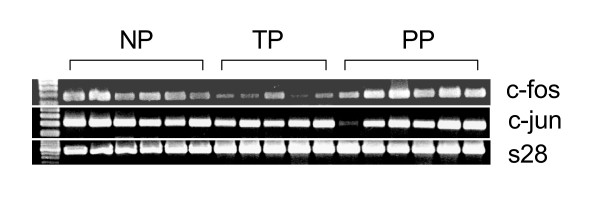
The presence of c-jun and c-fos mRNAs in 17 samples from the human uterine cervix as detected by RT-PCR analysis. Non-pregnant (NP, n = 6), term pregnant (TP, n = 5) and postpartum (PP, n = 6) groups. 28S was used as an internal standard to enable the comparison of the amount of mRNA loaded to each well.

Figure 7. illustrates the presence of c-jun and c-fos mRNAs in human uterine cervix in term pregnancy and immediately after parturition. S28 was used as a standard to enable comparison of the amount of RNA loaded to each well.

## Discussion

This study demonstrates alterations in the expression and distribution of the phosphorylated, activated MAPK forms and in their intracellular target transcription factor AP-1 in human uterine cervix at term pregnancy and immediately after parturition. To our knowledge, this is the first study identifying pMAPK activation in human uterine cervix. Rusycky [24] was the first to report an increase in MAPK cascade in vivo in rat myometrium with advancing gestation, and a decrease immediately before birth. Otun and collaborators [25] have described significantly elevated expressions of p38MAPK and ERK1 in the upper uterine segment at term and during labor, whereas ERK2 expression remained unchanged.

Proinflammatory cytokines increase exponentially in the uterus and cervix at the time for parturition [1,8,26-28]. Mechanical stretch and hypoxia are physiological features in the uterine and cervical tissues during gestation, which escalate with labor [27]. Proinflammatory interleukin (IL)-1β and mechanical stretch act through MAPKs to increase inducible cyclooxygenase (COX)-2 and IL-8 expression in human uterine myocytes [27]. Evidence suggests that some effect of mechanical tension could be mediated through an increased IL-1β level [27]. The activation of MAPK signaling observed in the present study may therefore be caused by elevated proinflammatory cytokine levels, mechanical tension and hypoxia in the lower uterine segment.

A high density of p38MAPK was observed in vascular endothelium at term pregnancy. Previous studies have shown, that p38MAPK facilitates the adhesion of leukocytes to endothelial cell integrins, chemotaxis, leukocyte oxidative burst and release of IL-1β and elastase [14,28]. The increased endothelial p38MAPK expression in term pregnancy, therefore, indicates that p38MAPK facilitates leukocyte extravasation in the uterine cervix before parturition.

The glandular epithelium displayed a high immunostaining intensity particularly for pJNK and for p38MAPK, but without changes between the groups. Uterine glandular epithelium is known to be a source of cytokine synthesis [29]. The comparable pJNK and p38MAPK expressions could be due to the limited number of samples or, alternatively, depend on an unchanged rate of glandular epithelial pMAPK activation in cervical ripening at parturition.

Cervical stroma, which is composed of 70–80% fibrous connective tissue and 5–15% smooth muscle [30] displayed increased immunostaining for all pMAPKs at term pregnancy, and an elevated pERK1/2 expression immediately after parturition. The expressions of proinflammatory transcription factors AP-1 and NFκB are increased in the cervical stoma at the time for parturition [31]. MAPK cascades, and estrogens, activate AP-1 and NFκB [15,16,19,21,22]. MAPKs and NFκB also activate the prostaglandin synthases phospholipase (PL) A_2 _and inducible COX-2 [5,18,26,27]. The collagen digesting enzyme metalloproteinase (MMP)-1 gene contains an AP-1 response element in its promoter region [32]. Thus, activation of MAPKs and their targets AP-1 and NFκB in the uterine cervix could promote cervical ripening through an increased prostaglandin synthesis and an enhanced collagenolysis [6-8,26,27,31]. Nuclear ER mediated activation of MAPKs is unlikely. Despite an exponentially increased maternal plasma estradiol concentration reaching 200 nmol/L at term pregnancy, the total cervical nuclear ER level is markedly decreased, and inhibitory ERβ subtype enhanced, as compared to the non-pregnant state. This suggests that the nuclear ER mediated genomic response is very limited at parturition [7,12]. Our findings are in agreement with previous reports, which have showed that estrogens at high concentrations evoke early events such as inflammatory cell influx, activation of MAPK pathways, adenyl cyclase (AC) and protein kinase C (PKC) [15,33,34]. Estradiol at low concentrations, comparable to the circulating levels during the menstrual cycle, elicits nuclear ER genomic activation, which requires hours or days for maximal gene activation [15-17,21,22,34].

In conclusion, our observations indicate activation of MAPK cascades, and increased expressions of their targets transcription factors AP-1 and NFκB [31] in human uterine cervix before labor. These results suggest a role for MAPK phosphorylation in cervical ripening at parturition. The exact mechanism behind MAPK activation should be a topic for future studies.
